# Supplementary material for: The microRNA-210-Stathmin1 Axis Decreases Cell Stiffness to Facilitate the Invasiveness of Colorectal Cancer Stem Cells
Source: Cancers (Basel). 2021 Apr 12;13(8):1833. doi: 10.3390/cancers13081833 (PMC8069838; doi:10.3390/cancers13081833)
Supplement: Supplementary file 1 [file cancers-13-01833-s001.zip › Supplementary files/Cancers supplementary figure legends-R1 tracked changes.docx]

**SUPPLEMENTARY FIGURE LEGENDS**

**Supplementary Figure 1: Characterization of sphere-derived cancer stem cells (SDCSCs). (a)** RT-qPCR validation of stemness gene expression (embryonic stem cell genes: *NANOG* and *POU5F1*; intestinal stem cell genes: *LGR5* and CD44; CSC gene: *SNAI1*) in HT29- and HCT15-SDCSCs and their corresponding parental cells. **(b)**  GSEA showed the association between the HT29-SDCSC-up regulated gene signature and 244 CRC samples with recurrence (R) versus nonrecurrence (NR) or severe tumor staging (I & II vs. III & IV, GSE17538). HT29-SDCSC-up regulated gene signature, the top 500 genes upregulated ≥ 2-fold in HT29-sphere/parental cells (GSE14733). ES, enrichment score. NES, normalized enrichment score. **(c-d)** RT-qPCR examining the expression of miR-210-3p in SW1116 and Colo205 CRC cells. (c) and in primary and liver metastatic PDXs (d). PDX, patient-derived xenografts. Unless otherwise stated, all data in bar charts are expressed as the mean ± SD values. **p* < 0.05; ***p* < 0.01; ****p* < 0.001 (Student’s t-test).

**Supplementary Figure 2: Silencing miR-210-3p expression does not affect the symmetric cell division (SCD) of HT29-SDCSCs. (a)** A flow chart depicting the procedures for thymidine-nocodazole-blebbistatin synchronization in HT29-SDCSCs. SCM, stem cell medium. **(b)** RT-qPCR validation of miR-210-3p expression in HT29-SDCSCs receiving control antagomir (Scramble) or miR-210-3p antagomir (Antagomir-210-3p). Cholesterol-modified antagomirs were added at a concentration of 50 nM. **(c)** Histograms showing the percentages of symmetric and asymmetric BrdU segregation in the indicated HT29-SDCSCs cultured in stem cell medium. The number of paired cells analyzed is noted in the brackets. The *p* values were estimated by the χ2 test. **(d)** Representative images of symmetric and asymmetric BrdU segregation in HCT15-SDCSCs. BrdU, green; DNA, blue; SCD, symmetric division; ACD, asymmetric division. Scale bars, 10 μm. **(e)** RT-qPCR examining the expression of stemness and differentiation markers in CRC cells. ns, nonsignificant. Data in bar charts are expressed as the mean ± SD values.

**Supplementary Figure 3: Restoration of STMN1 does not consistently alter the expression of epithelial and mesenchymal markers in HT29- and HCT15-SDCSCs. (a-c)** RT-qPCR examining the expression of an epithelial cell marker, CDH1, (a), a mesenchymal marker, *CDH2* (b), and a mesenchymal marker, *VIM* (c) in control SDCSCs (Vec) and SDCSCs ectopically expressing STMN1 (STMN1 Myc-DDK). ns, nonsignificant. Data are expressed as the mean ± SD values. **p* < 0.05; ***p* < 0.01; ****p* < 0.001 (Student’s t-test).

**Supplementary Figure 4: Ectopically expression of STMN1 reverses the miR-210-regulated trans well migration ability and elasticity of HT29 parental cells. (a)** RT-qPCR examining the expression of miR-210-3p at indicated CRC cells. **(b)** Western blot showing the expression of Myc-DDK-tagged STMN1 by an anti-FALG antibody at indicated CRC cells. **(c-d)** The relativity viability (c) and clonogenicity (d) of indicated CRC cells. **(e)** Representative images showing the colonies generated. **(f-h)** Histograms showing the relative transwell migration ability (f), intracellular elasticity (g) and expression of EMT markers (*CDH1*, *CDH2* and *VIM*) (h) of indicated cells. ns, nonsignificant. Data are expressed as the mean ± SD values. **p* < 0.05; ****p* < 0.001 (Student’s t-test).

**Supplementary Figure 5: Overexpression of STMN1 inhibits the transwell migration potential and promotes intracellular elasticity of HT29 parental cells. (a)** Western blots showing the expression of STMN1 in control HT29 cells (Vec) and HT29 cells ectopically expressing STMN1. An anti-FLAG antibody was used to detect the expression of exogenous STMN1 with the Myc-DDK tag. **(b)** Relative viability of cells as assessed by an MTT assay. ns, nonsignificant. **(c)** The colony formation of control (Vec) and STMN-expressing (STMN1 DKK-Myc) HT29 cells. ns, nonsignificant. **(d)** Representative images showing the colonies generated. **(e)** RT-qPCR validation of the expression of EMT markers (CDH1, CDH2 and VIM) in the established HT29-Vec and HT29 STMN1-Myc-DDK cells. **(f-g)** Histograms showing the relative transwell migration ability (f) and intracellular elasticity (g) of indicated cells. ns, nonsignificant. Data in bar charts are expressed as the mean ± SD values. **p* < 0.05; ***p* < 0.01***; *p* < 0.001 (Student’s t-test).

**Supplementary Figure 6: Decreased expression of STMN1 in liver metastatic CRC specimens. (a)** Representative images illustrating STMN1 immunoreactivity in paired primary and metastatic CRC specimens. **(b)** Histogram showing the histological scores (H-scores) of CRC specimens (n=11). ***p* < 0.01 (Student’s t-test). **(c)** The bar charts showing the expression of STMN1 in liver and lymph node metastatic CRC specimens deposited at GSE26571 retrieved from the Human Cancer Metastasis Database (HCMDB). The box plots show the sample maximum (upper end of the whisker), upper quartile (top edge of the box), median (band in the box), lower quartile (bottom edge of the box), and sample minimum (lower end of the whisker) values. **(d)** The bar charts showing the expression of STMN1 in liver and lung metastatic CRC specimens deposited at GSE68468 retrieved from the Human Cancer Metastasis Database (HCMDB). The box plots show the sample maximum (upper end of the whisker), upper quartile (top edge of the box), median (band in the box), lower quartile (bottom edge of the box), and sample minimum (lower end of the whisker) values.

**Supplementary Figure 7: Examining the expression of miR-210-3p-STMN1 axis components in Snail-expressing HCT15 cells. (a)** RT-qPCR validation of the expression of *SNAI1* and *CDH1* in established stable HCT15-Vec and HCT15-Snail cells. **(b)** RT-qPCR validation of the expression of *miR-210-3p* and STMN1 in established stable HCT15-Vec and HCT15-Snail cells. Data are expressed as the mean ± SD values. ****p* < 0.001 (Student’s t-test).

**Supplementary Figure 8: Uncropped blots.**

**Supplementary Video 1:** **A video showing the Brownian motion of intracellular fluorescent beads in HCT15 cells and HCT15-SDCSCs.**
