# Supplementary material for: The microRNA-210-Stathmin1 Axis Decreases Cell Stiffness to Facilitate the Invasiveness of Colorectal Cancer Stem Cells
Source: Cancers (Basel). 2021 Apr 12;13(8):1833. doi: 10.3390/cancers13081833 (PMC8069838; doi:10.3390/cancers13081833)

Figure S8

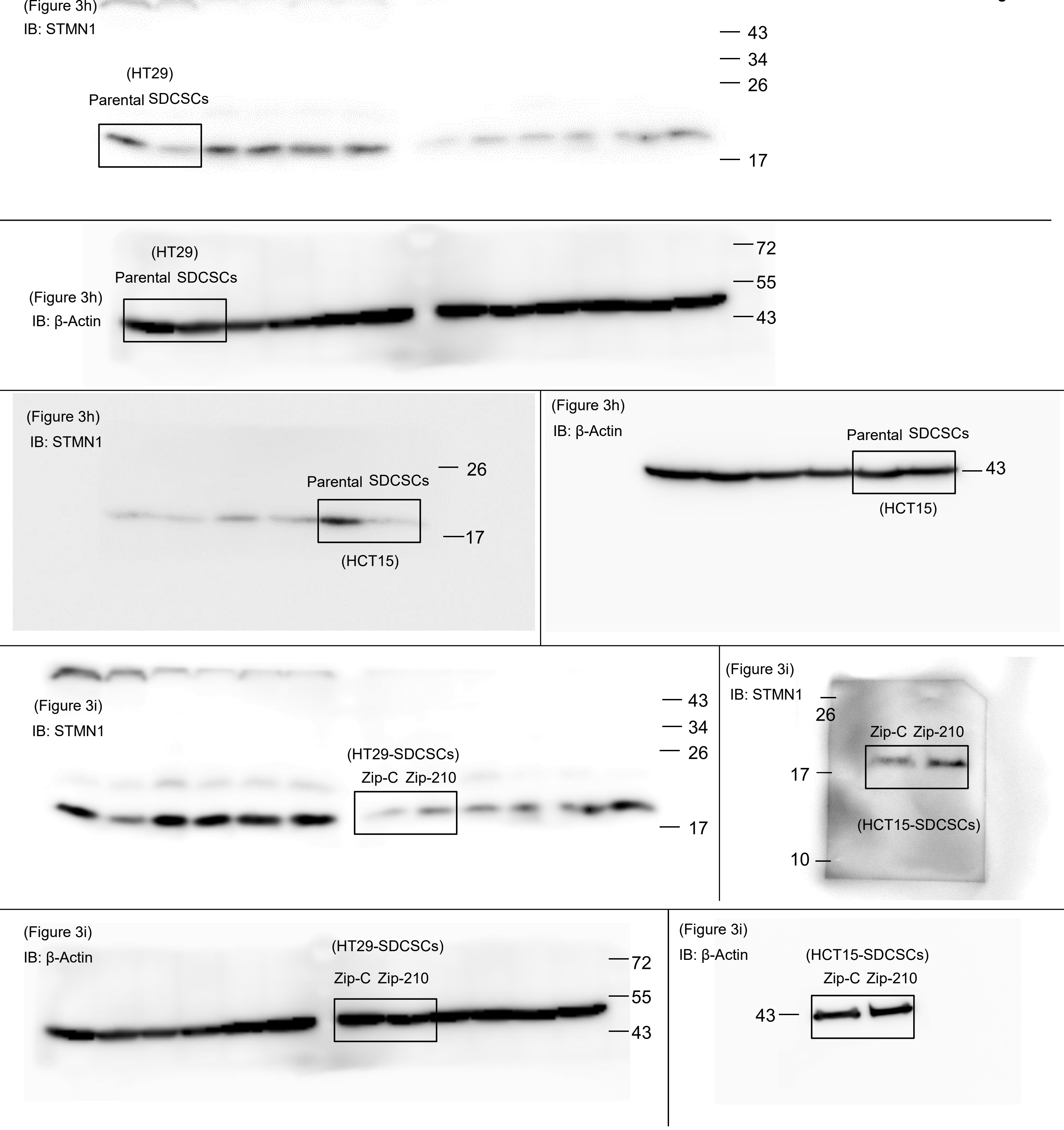
(Figure 3h)
IB: STMN1
(HT29)
Parental SDCSCs
43
34
26
17
(HT29)
Parental SDCSCs
(Figure 3h)
IB: β-Actin
72
55
43
(Figure 3h)
IB: STMN1
Parental SDCSCs
26
17
(HCT15)
(Figure 3h)
IB: β-Actin
Parental SDCSCs
43
(HCT15)
(Figure 3i)
IB: STMN1
43
34
26
17
(HT29-SDCSCs)
Zip-C Zip-210
(Figure 3i)
IB: STMN1
26
Zip-C Zip-210
17
(HCT15-SDCSCs)
10
(Figure 3i)
IB: β-Actin
(HT29-SDCSCs)
Zip-C Zip-210
72
55
43
(Figure 3i)
IB: β-Actin
(HCT15-SDCSCs)
Zip-C Zip-210
43

Figure S8

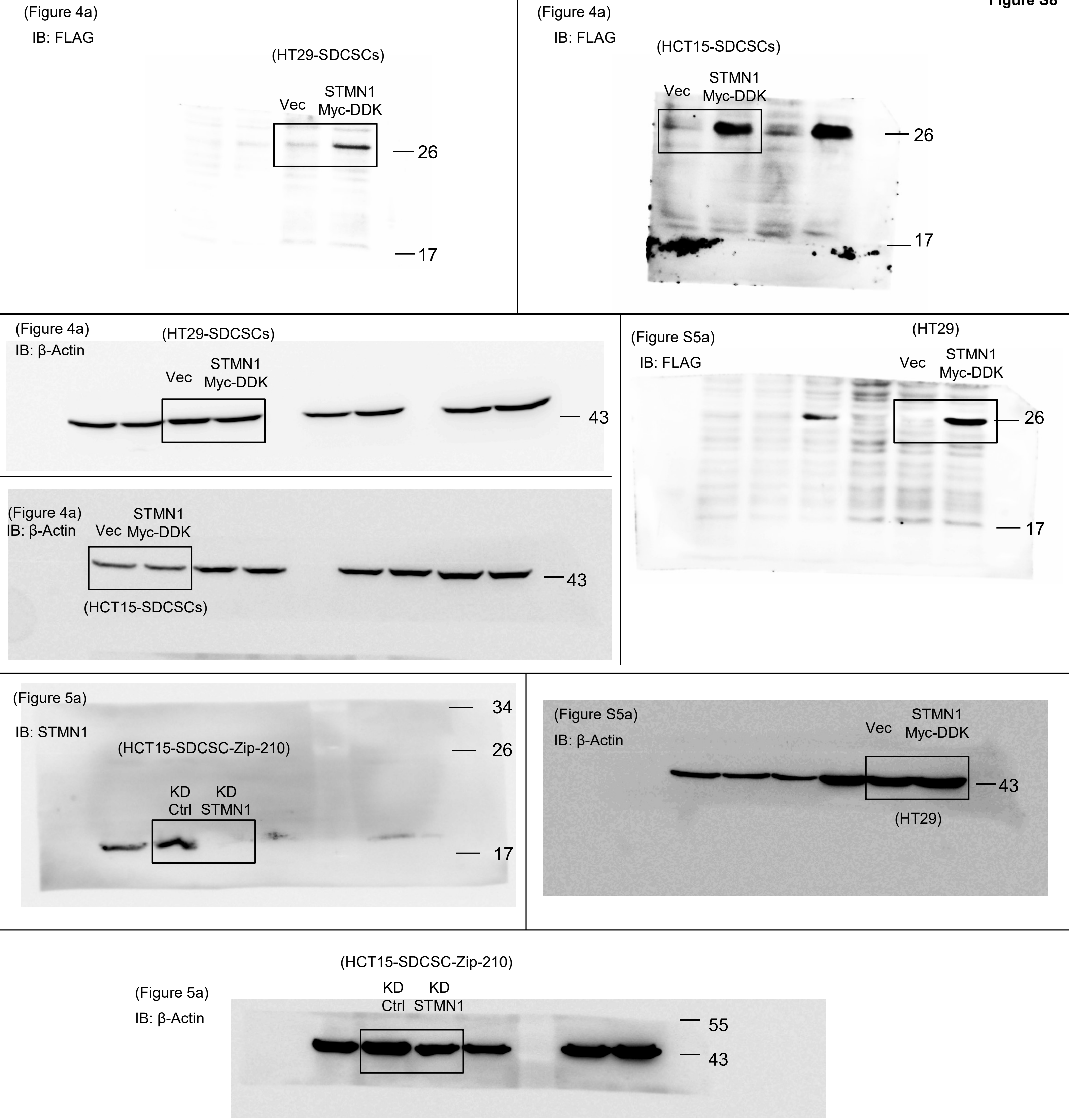

(Figure 4a)
IB: FLAG
(HT29-SDCSCs)
Vec
STMN1
Myc-DDK
26
17
(Figure 4a)
IB: FLAG
(HCT15-SDCSCs)
Vec
STMN1
Myc-DDK
26
17
(Figure 4a)
IB: β-Actin
(HT29-SDCSCs)
Vec
STMN1
Myc-DDK
43
(Figure 4a)
IB: β-Actin
Vec
STMN1
Myc-DDK
(HCT15-SDCSCs)
43
(Figure S5a)
IB: FLAG
(HT29)
Vec
STMN1
Myc-DDK
26
17
(Figure 5a)
IB: STMN1
(HCT15-SDCSC-Zip-210)
KD
Ctrl
KD
STMN1
34
26
17
(Figure S5a)
IB: β-Actin
Vec
STMN1
Myc-DDK
43
(HT29)
(HCT15-SDCSC-Zip-210)
(Figure 5a)
IB: β-Actin
KD
Ctrl
KD
STMN1
55
43

Figure S8

(Figure 3f)

IB: STMN1

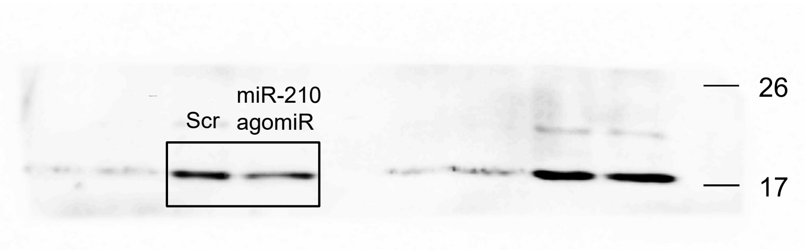

(Figure 3f)

IB: β-Actin

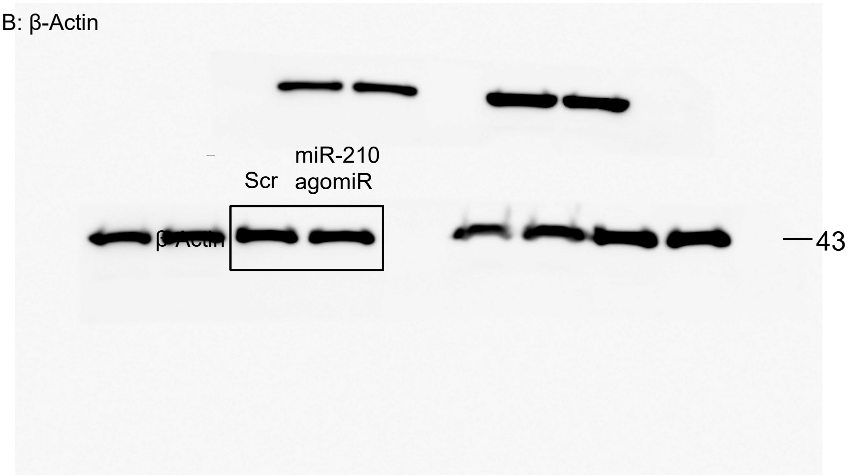

(Figure S4b)

IB: FLAG

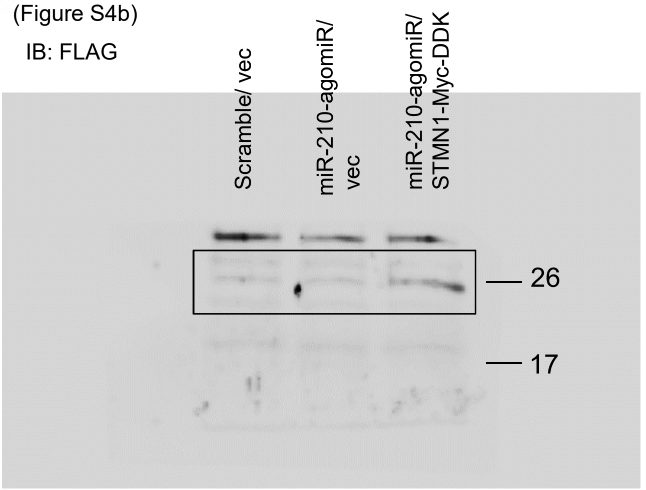

(Figure S4b)

IB: β-Actin

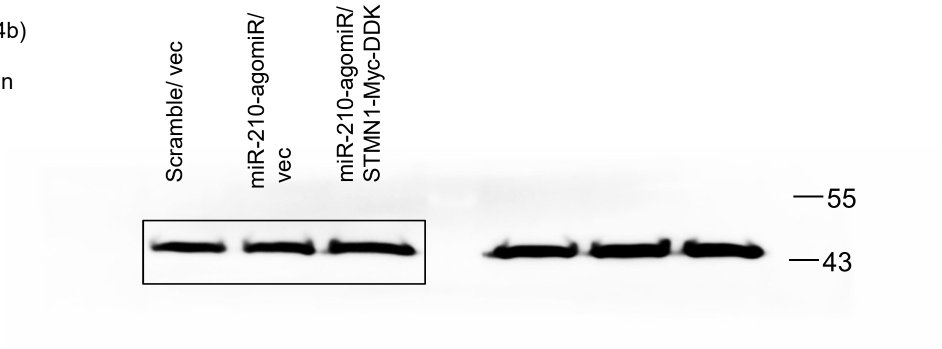

Supplement: Supplementary file 1 [file cancers-13-01833-s001.zip › Supplementary files/Figure S8-western blot images update.pdf]
