# Supplementary figures and images for: The microRNA-210-Stathmin1 Axis Decreases Cell Stiffness to Facilitate the Invasiveness of Colorectal Cancer Stem Cells
Source: Cancers (Basel). 2021 Apr 12;13(8):1833. doi: 10.3390/cancers13081833 (PMC8069838; doi:10.3390/cancers13081833)

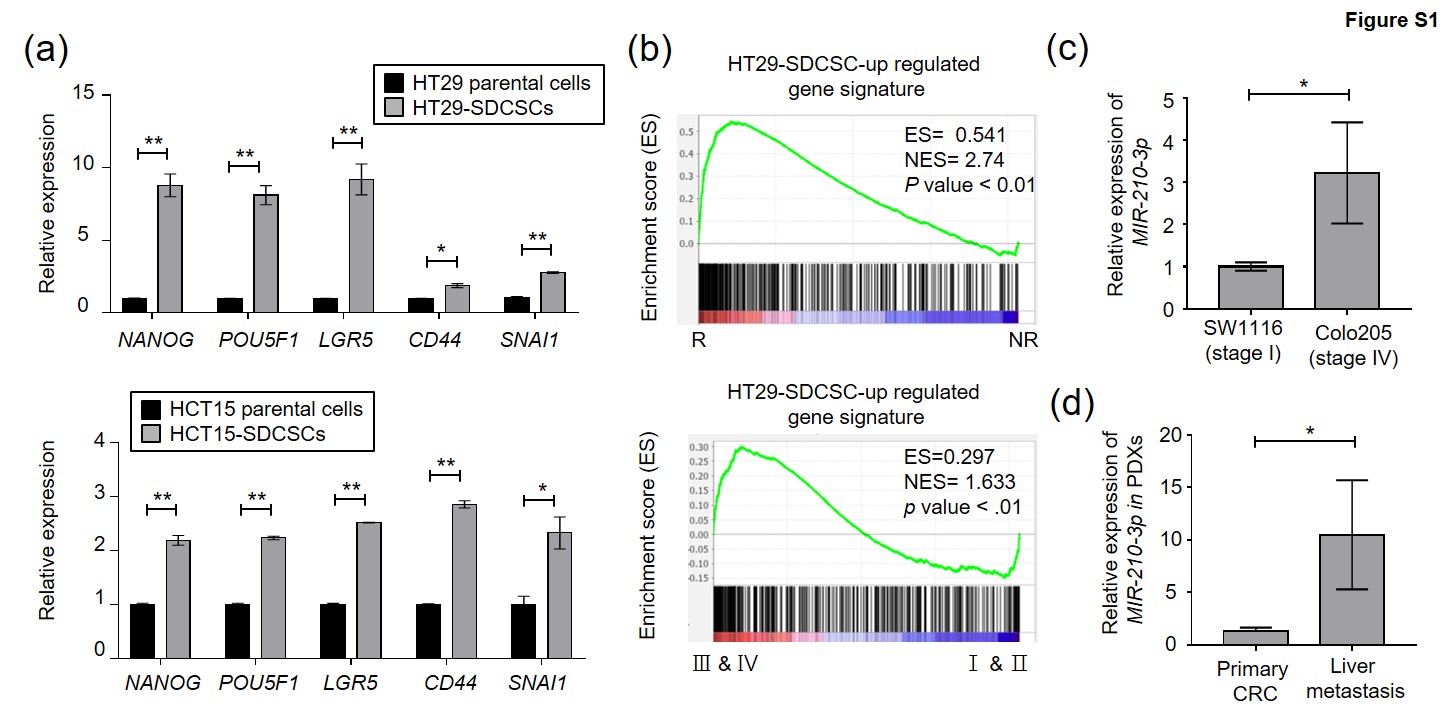

Supplement: Supplementary file 1 [file cancers-13-01833-s001.zip › Supplementary files/Figure S1.jpg]

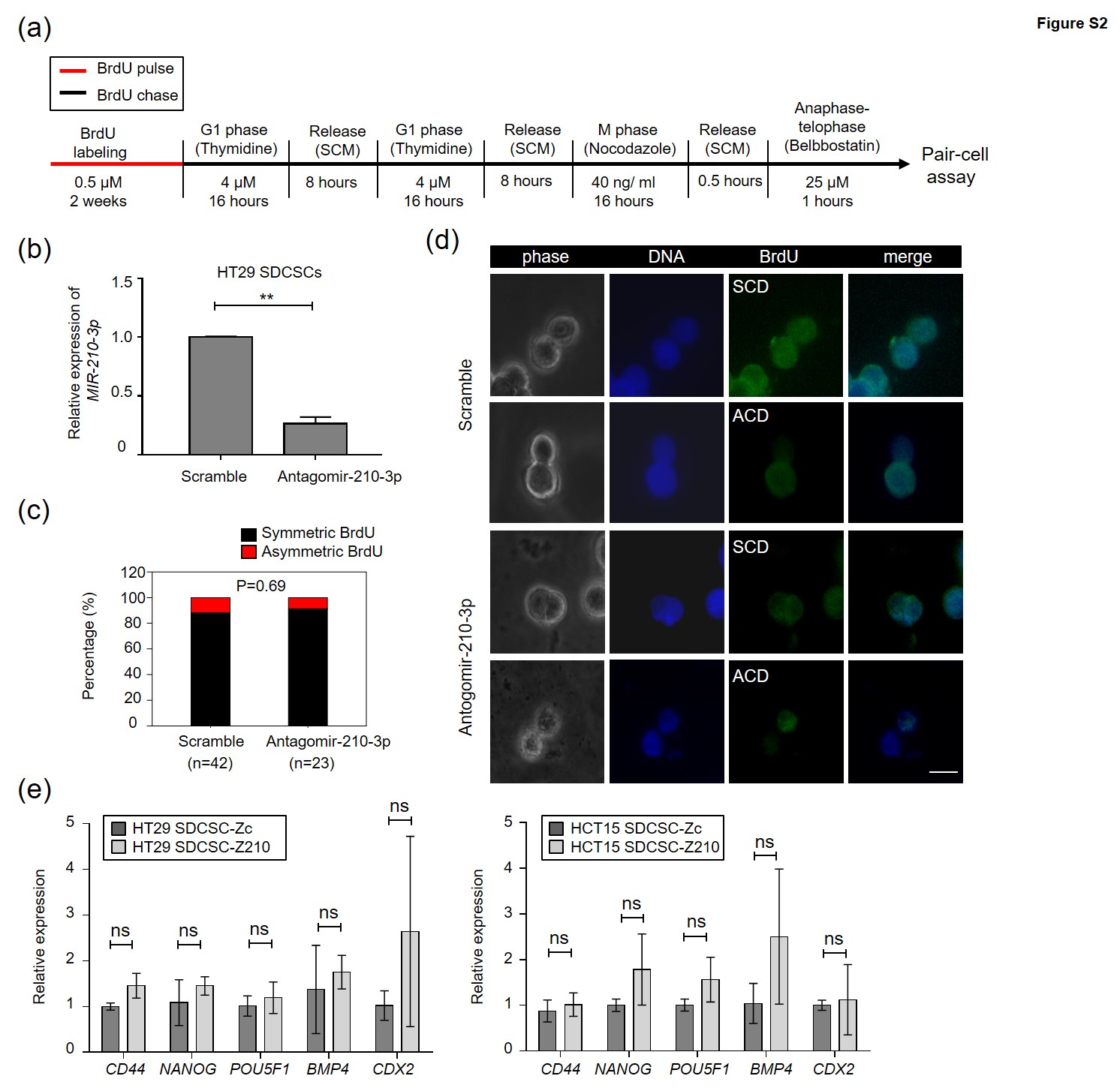

Supplement: Supplementary file 1 [file cancers-13-01833-s001.zip › Supplementary files/Figure S2-revised.jpg]

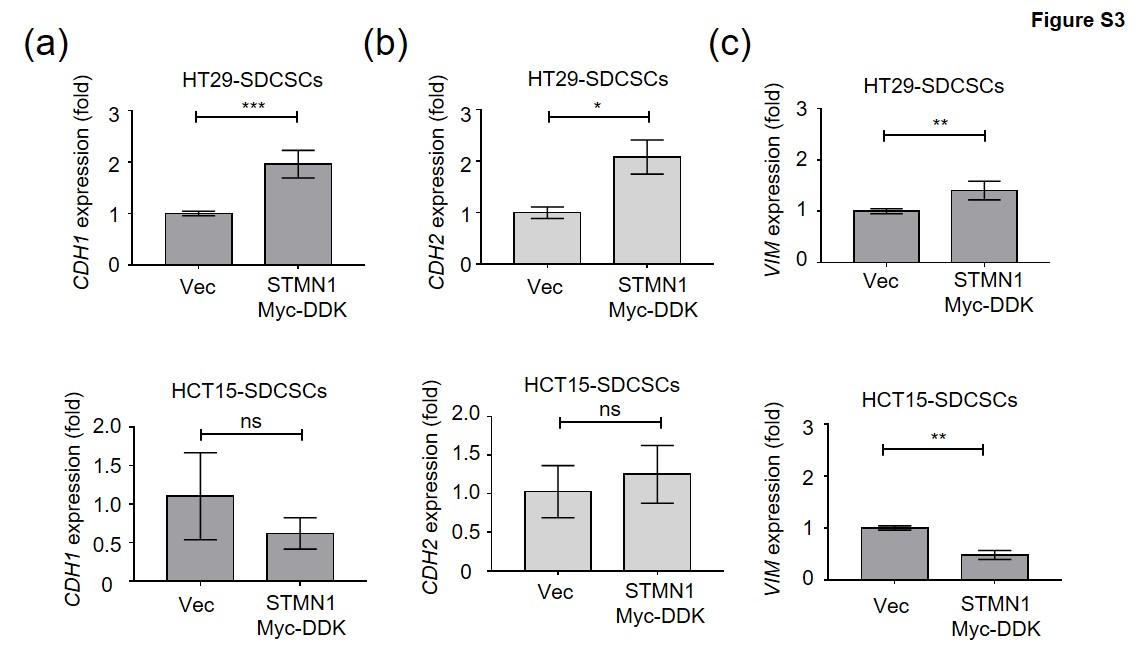

Supplement: Supplementary file 1 [file cancers-13-01833-s001.zip › Supplementary files/Figure S3-revised.jpg]

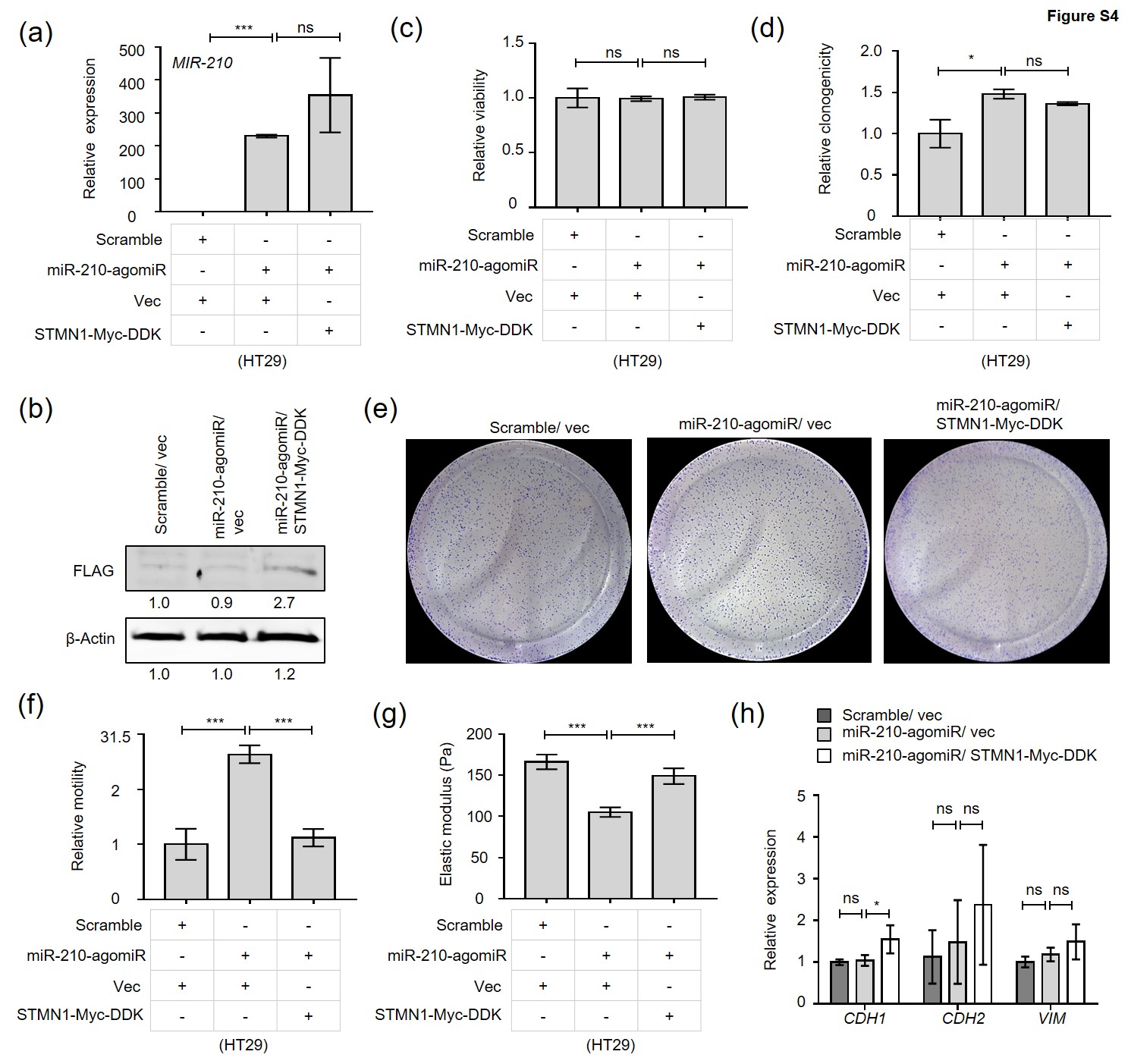

Supplement: Supplementary file 1 [file cancers-13-01833-s001.zip › Supplementary files/Figure S4-revised.jpg]

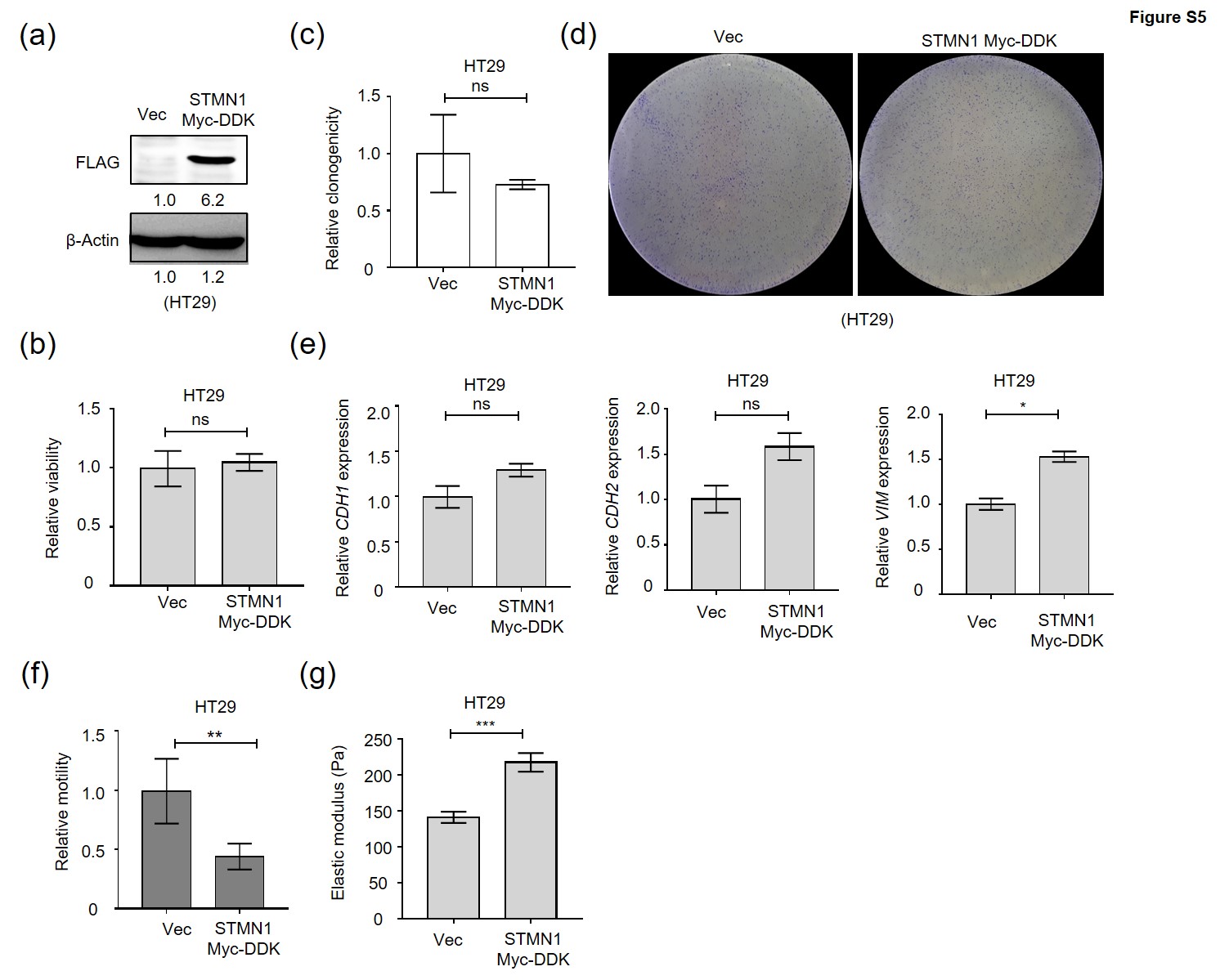

Supplement: Supplementary file 1 [file cancers-13-01833-s001.zip › Supplementary files/Figure S5-revised.jpg]

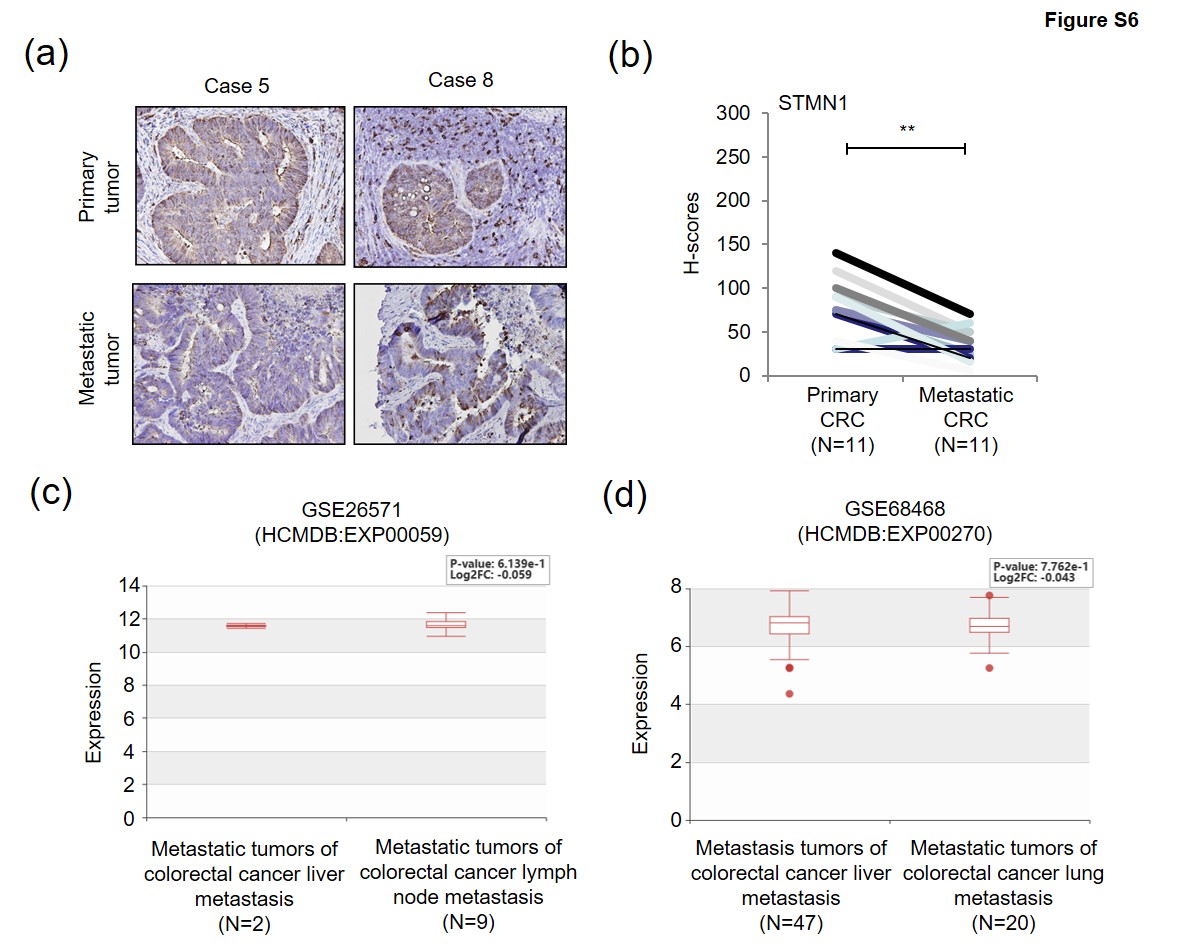

Supplement: Supplementary file 1 [file cancers-13-01833-s001.zip › Supplementary files/Figure S6-revised.jpg]

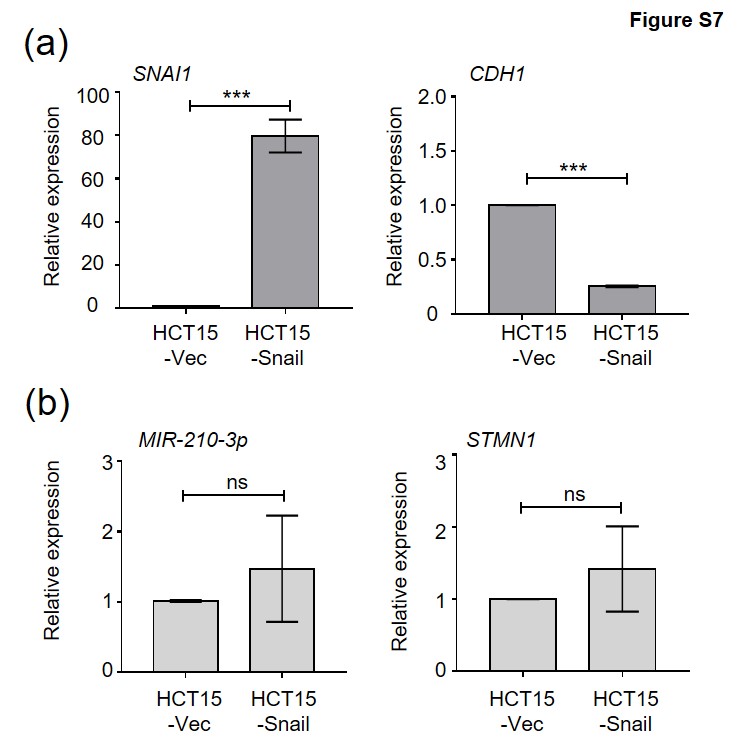

Supplement: Supplementary file 1 [file cancers-13-01833-s001.zip › Supplementary files/Figure S7.jpg]
